# Effects of Different Grazing Intensities on Grassland Production in China: A Meta-Analysis

**DOI:** 10.1371/journal.pone.0081466

**Published:** 2013-12-06

**Authors:** Liang Yan, Guangsheng Zhou, Feng Zhang

**Affiliations:** 1 State Key Laboratory of Vegetation and Environmental Change, Institute of Botany, Chinese Academy of Sciences, Beijing, China; 2 Chinese Academy of Meteorological Sciences, Beijing, China; 3 University of Chinese Academy of Sciences, Beijing, China; Beijing Forestry University, China

## Abstract

**Background:**

Grazing is one of the main grassland disturbances in China, and it is essential to quantitatively evaluate the effects of different grazing intensities on grassland production for grassland carbon budget and sustainable use.

**Methods:**

A meta-analysis was conducted to reveal general response patterns of grassland production to grazing in China. We used weighted log response ratio to assess the effect size, and 95% confidence intervals to give a sense of the precision of the estimate. Grazing effects were estimated as a percentage change relative to control (%).

**Results:**

A total of 48 studies, including 251 data sets, were included in the meta-analysis. Grazing significantly decreased total biomass by 58.34% (95% CI: −72.04%∼−37.94%, CI: Confidence Interval), increased root/shoot ratio by 30.58% and decreased litter by 51.41% (95% CI: −63.31%∼−35.64%). Aboveground biomass and belowground biomass decreased significantly by 42.77% (95% CI: −48.88%∼−35.93%) and 23.13% (95% CI: −39.61%∼−2.17%), respectively. However, biomass responses were dependent on grazing intensity and environmental conditions. Percentage changes in aboveground biomass to grazing showed a quadratic relationship with precipitation in light grazing intensity treatment and a linear relationship in moderate and heavy grazing intensity treatment, but did not change with temperature. Grazing effects on belowground biomass did not change with precipitation or temperature. Compared to the global average value, grazing had greater negative effects on grassland production in China.

**Conclusions:**

Grazing has negative effects on grassland biomass and the grazing effects change with environmental conditions and grazing intensity, therefore flexible rangeland management tactics that suit local circumstances are necessary to take into consideration for balancing the demand of grassland utilization and conservation.

## Introduction

Grasslands in China occupy approximately 4×10^8^ ha, accounting for 41% of land surface [1], [2]. Grazing is one of the most important disturbances of grasslands in terms of both grassland production and vegetation dynamics [3], [4], [5]. Nearly 100% of grasslands in China are grazed, grassland deterioration is severe. The increasing human and livestock populations caused dust storms in past decade. Considering sustainable development of grassland, the government has put great efforts to deal with grassland degradation, increased funding and research initiatives to seek effective management practices for sustaining grasslands in China [6]. Considerable studies were conducted to investigate grazing effects on grassland production, including grassland total biomass [7], [8], aboveground biomass [9], [10], [11], belowground biomass [12], [13], litter [14], [15] and root/shoot ratio [16], [17], [18]. These studies contributed a lot to understand impacts of grazing on grasslands, but most of these studies were limited to site scale. Some of these studies proposed that grazing had positive effects on grassland production by compensatory growth [19], [20]. Other studies, however, showed that grazing had significantly negative effects on grassland and might cause grassland degradation [21], [22]. Moreover, the effects of grazing on grassland production are affected by environmental conditions, grazing intensities of each research site. These seemingly contradictory results suggest that it’s necessary to integrate site studies along a broad range of environments and different grazing intensities to further explore the quantitative effects of grazing on grasslands.

Meta-analysis is the quantitative synthesis, analysis and summary of a collection of studies, which has been proven to be a powerful statistical tool and widely used in ecological research [23], [24], [25], [26], [27], [28]. Milchunas & Lauenroth (1993) compield 276 data sets from studies around to world to quantitatively assess grazing impacts on grassland ecosystems. For the entire data set, grazing decreased aboveground net primary production by 23% and increased belowground biomass by 20%. The negative effects of grazing on aboveground production were compensated for almost the same amount of positive responses of belowground biomass. This study of Milchunas & Lauenroth (1993) provided general patterns of biomass response to grazing at global scale. Furthermore, grazing evolutionary history plays a key role in grasslands’ response to grazing and will determine present ecological process [29], [30], [31], [32]. Compared to other regions of the world, grasslands in China are grazed thousands years ago and experienced drastic land intensification due to population increases and settlement [33] in recent years. The longer and more intensive grazing evolutionary history may change response patterns of grassland production to current grazing. To date, the effects of different grazing intensities on grassland production have not been evaluated in China as a whole, which limits our understanding of the grazing effects on grasslands and sustainable management of grasslands in China.

The effect of grazing on grassland production in China depended on the research object (aboveground biomass, belowground biomass or total biomass), scale (species or community), environmental conditions (dry area or humid area), experimental design (light, moderate or heavy grazing intensity) and grazing evolutionary history of the site [30], [34], [35].

The objective of this paper is to quantitatively assess grazing effects on China’s grassland production (including total biomass, aboveground biomass, belowground biomass, litter and root/shoot ratio) based on existing data sets from publications and a transect survey. The aims of this synthesis were to test the following hypotheses:

1. Responses of grassland production to grazing in China varied with grazing intensity and environmental conditions of the site;

2. Aboveground biomass was generally expected to increase under light grazing intensity, but to decrease under moderate and heavy grazing intensities;

3. Belowground biomass increased due to biomass allocated more to belowground in grazed sites, with a higher root/shoot ratio. However, it may decrease in long and heavy grazing intensity treatment;

4. Total biomass may not significantly change at light and middle grazing intensity, and decreased at high grazing intensity;

5. Litter was generally decreased by grazing.

## Materials and Methods

### Study Site

Grasslands in China range from the high-altitude of the Qinghai-Tibetan plateau to the low-altitude steppes of Inner Mongolia.The grassland ecosystems in China are classified into four major types [6], [36]: typical steppes, meadow steppes, desert steppes and alpine steppes.

Typical steppes occur on a semi-arid climate in the temperate zone with annual precipitation of about 350 mm and the perennial grasses are drought tolerant. Meadow steppes are developed in the most moist and fertile areas with annual precipitation of 450 mm. Desert steppes are found in arid areas, its annul precipitation is less than 250 mm [6]. Alpine steppes are distributed between 3200 and 5200 m height above sea level [36], with annual precipitation varies from about 600 mm in the east to under 60 mm in the west [37].

The grasslands in China are used to graze domestic livestock such as sheep, goats, cattle, and yaks. Grasslands are major sources of livestock products such as mutton, milk, sheep wool, goat wool and cashmere for people.

### Data Sources and Compilation

#### 1. Data from literature

**Figure 1:**
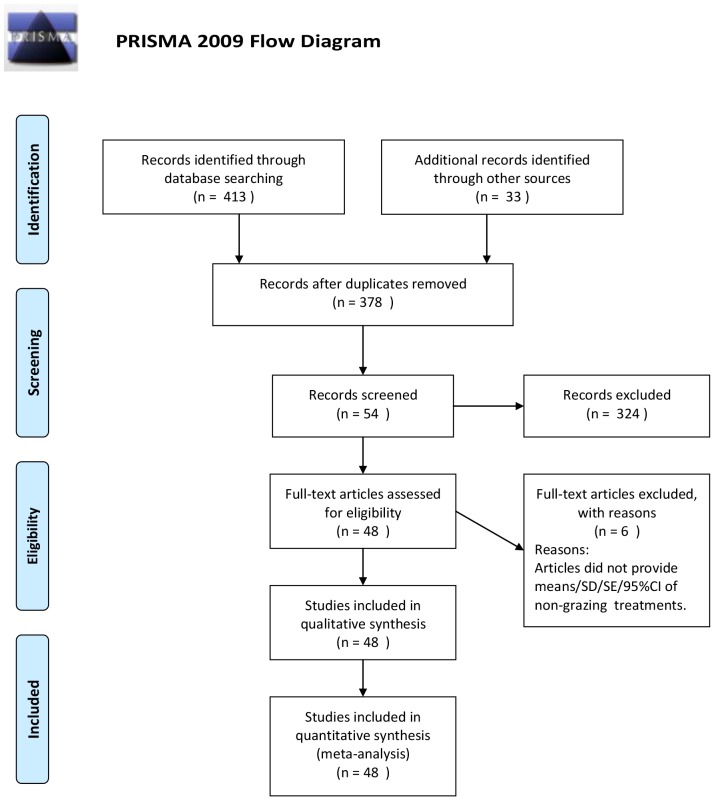
The flow diagram.

We built a database by searching studies in Web of Science and China National Knowledge Infrastructure. Grazing, grassland, total biomass, aboveground biomass, belowground biomass, root/shoot ratio, carbon allocation, biomass allocation, litter were used as keywords in the searching process (Figure 1). We extracted data directly from tables or text in literatures or indirectly from figures using GetData Graph Digitizer 2.22 (http://getdata-graph-digitizer.com). The following criteria were used to include papers in our analysis:

1. The study was carried out in grassland ecosystems in China;

2. At least one variable, including total biomass, aboveground biomass, belowground biomass, root/shoot ratio, litter was reported;

3. Means, standard errors, standard deviations or confidence intervals, sample sizes of both control and grazing treatments were available;

4. Grazing intensity and grassland type were provided.

Biomass and litter units were converted to g/m^2^ where necessary, other variables such as latitude, longitude, altitude, mean annual temperature and mean annual precipitation were also collected for further analysis.

#### 2. Data from the transect survey

**Figure 2:**
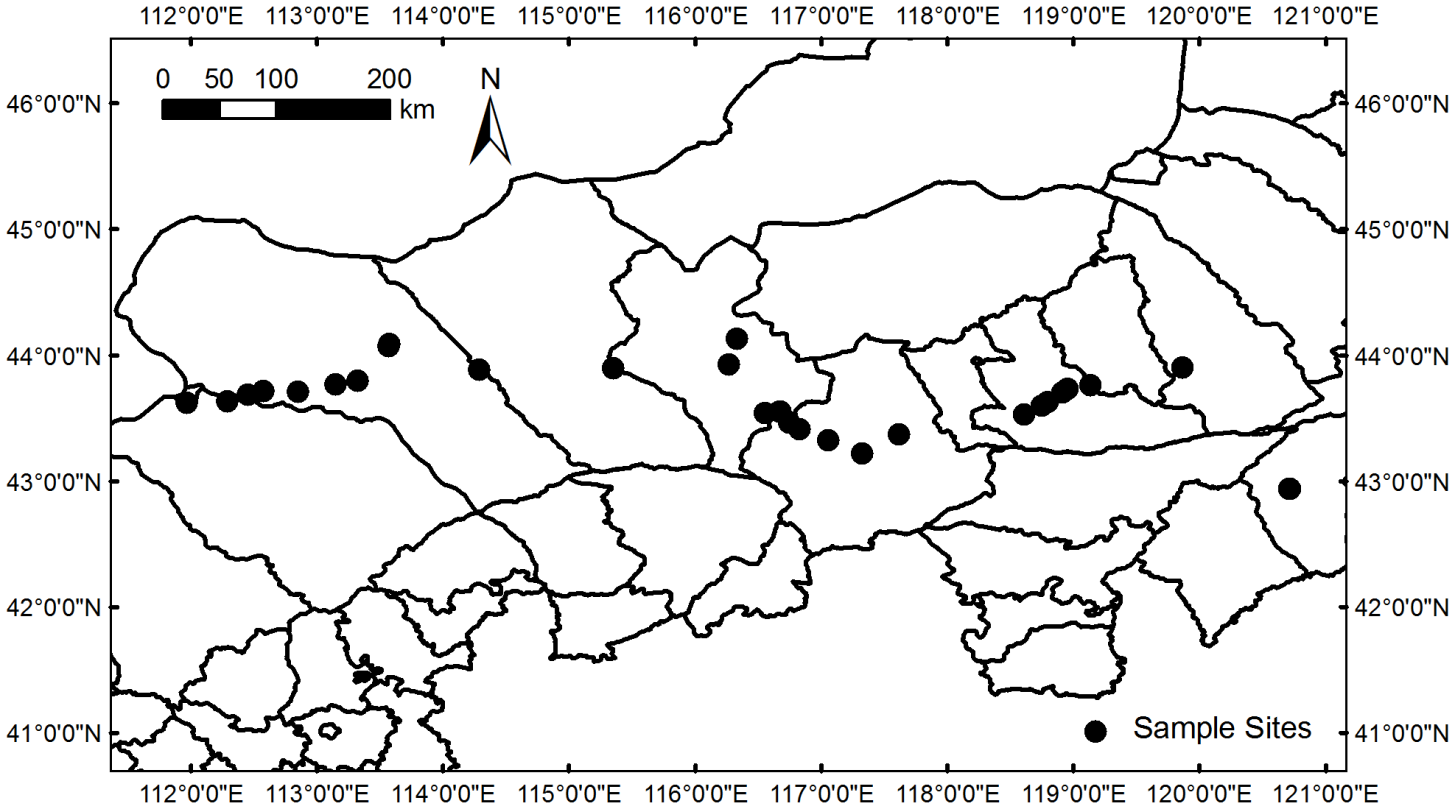
Map of sample sites (solid circles) in the transect survey.

The grassland transect survey was conducted from August to September in 2009, from Erenhot in the west to Tongliao city in the east across Inner Mongolia (Figure 2). This area includes three types of grasslands: meadow steppe, typical steppe, and desert steppe. We selected 28 sites across this transect. For each site, we collected three 50 cm×50 cm quadrats for aboveground biomass and litter. Belowground biomass was collected using five 10 cm diameter large cores and separated into 0∼10 cm, 10∼20 cm and 20∼30 cm depth. Land use history and current grazing intensity were collected from research site or estimated based on information from local herdsman or farmer. Biomass samples were returned to State Key Laboratory of Vegetation and Environmental Change, Institute of Botany, the Chinese Academy of Sciences. The samples were dried at 70°C for 48 hours and weighted in the lab.

A total of 48 studies including 251 sets of data were included in our analysis, including 12 sets of total biomass, 80 sets of aboveground biomass and 108 sets of belowground biomass. Data sets of litter and root/shoot ratio were 16 and 35, respectively. The transect data accounted for 40% of all data. Of the whole data sets, the latitude ranged from 25°48′ to 49°22′N and longitude ranged from 101°12′ to 131°14′E, altitude ranged from 55 to 3500 m, grazing intensity ranged from light, moderate or heavy treatments, grassland type ranged from typical steppe, meadow steppe, alpine steppe or desert steppe.

#### 3. Environmental data

Grazing has varying effects in different environments. Temperature and precipitation are key environmental factors affecting grass production. Compared to mean annual temperature and mean annual precipitation, temperature and precipitation of experimental year are more suitable to represent heat and moisture conditions of plant growth. Grazer effects may shift from negative to positive with increasing precipitation or temperature. Thus, we interpolated temperature and precipitation data sets using ANUSPLIN 4.3 [38] based on shared data from China Meteorological Data Sharing Service System. Then we prepared temperature and precipitation data sets for both experiments from literatures and transect survey to further explore relationships between environmental factors and response patterns of grasslands to grazing. The data was prepared according to the location and experiment time of each study.

### Meta-analysis

Meta-analysis standardizes results across many studies by calculating effect size and thus makes the results of studies comparable. Effect size has various calculation methods, of which response ratio is widely used. It’s the ratio of mean in treatment group (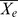) to that of the control group (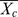) and converted to the metric of natural log [39]:
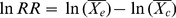
(1)


The weighting factor of each study was estimated by:
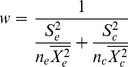
(2)where 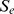 and 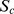 are standard deviations for the treatment and control groups, respectively; 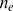 and 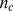 are sample sizes for the treatment and control groups, respectively. Meta-analysis gives greater weight to studies whose estimates have greater precision so the power of the tests will increase by calculating a weighted log response ratio (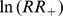) according to individual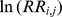:
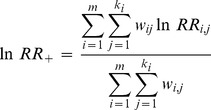
(3)where, 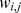 is the weighting factor of each group, 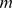 is the number of groups and 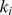 is the number of comparisons in the *i*th group. Standard error (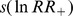) is calculated as:
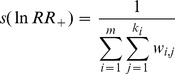
(4)with 95% confidence interval for the log response ratio is:



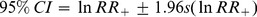
(5)The total heterogeneity (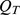) of effect sizes among studies included within-group (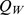) and between-group (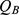) heterogeneity:
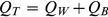
(6)


The total heterogeneity is calculated as follow:
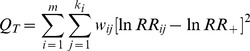
(7)with degrees of freedom (df): 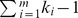. The within-group (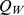) heterogeneity is estimated by:
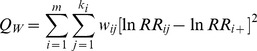
(8)with df = (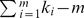). The between-group (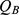) heterogeneity is calculated as:
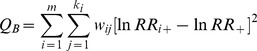
(9)with df = 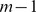. If 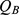 is larger than a critical value, the independent variable has a significant influence on the response ratio [40]. Statistical significance was tested at *P*<0.05 level.

Data analysis was conducted by using Metawin2.0 [41] and figures were drawn in Sigmaplot11.0 (http://www.sigmaplot.com). Grazing effects were estimated as a percentage change relative to control (%):
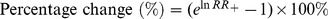
(10)


Grazing was considered to have a significant influence on a variable if the bootstrap CI of its percentage changes did not overlap zero. Response of grass biomass in a certain grazing intensity treatment was considered to significantly different if their bootstrap CIs did not overlap [25], [39].

## Results

### General Response Patterns of Grassland Production to Grazing

**Figure 3:**
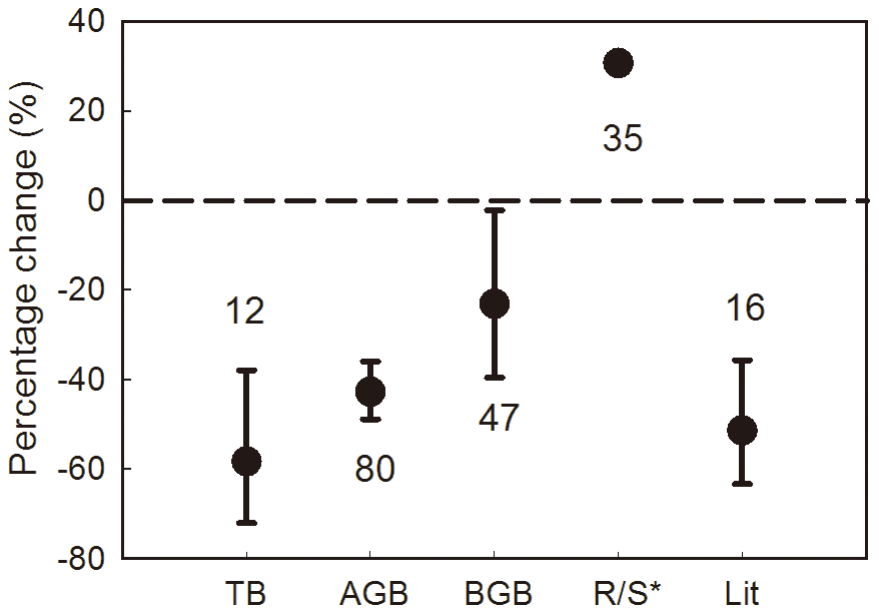
Responses of grassland production to grazing as a percentage change relative to control (%). Total biomass, aboveground biomass (AGB), belowground biomass (BGB), root/shoot ratio and litter were included in the meta-analysis. Values are means ± 95% CI and numbers of observations are shown near the bar. *The confidence intervals for the root/shoot ratio could not be calculated for the most root/shoot ratio data we collected did not have the “SD” or “SE”, but the root/shoot ratio is an essential part of grassland production, thus we still included it.

Total biomass was significantly reduced by 58.34% (95% CI: −72.04%∼−37.94%). However, the root/shoot ratio increased by 30.58% in grazing treatment. Aboveground and belowground biomass was significantly reduced by 42.77% (95% CI: −48.88%∼−35.93%) and 23.13% (95% CI: −39.61%∼−2.17%), respectively. Meanwhile, grazing significantly reduced litter by 51.41% (95% CI: −63.31%∼−35.64%) (Figure 3).

### Response Patterns of Aboveground Biomass to Grazing

The response patterns of aboveground biomass to grazing may vary with grassland types and grazing intensity. However, there was no significant difference among grassland types (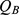 = 4.69, P = 0.19), but grazing intensity had a significant effects on aboveground biomass response patterns (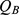 = 100.09, P<0.001).

**Figure 4:**
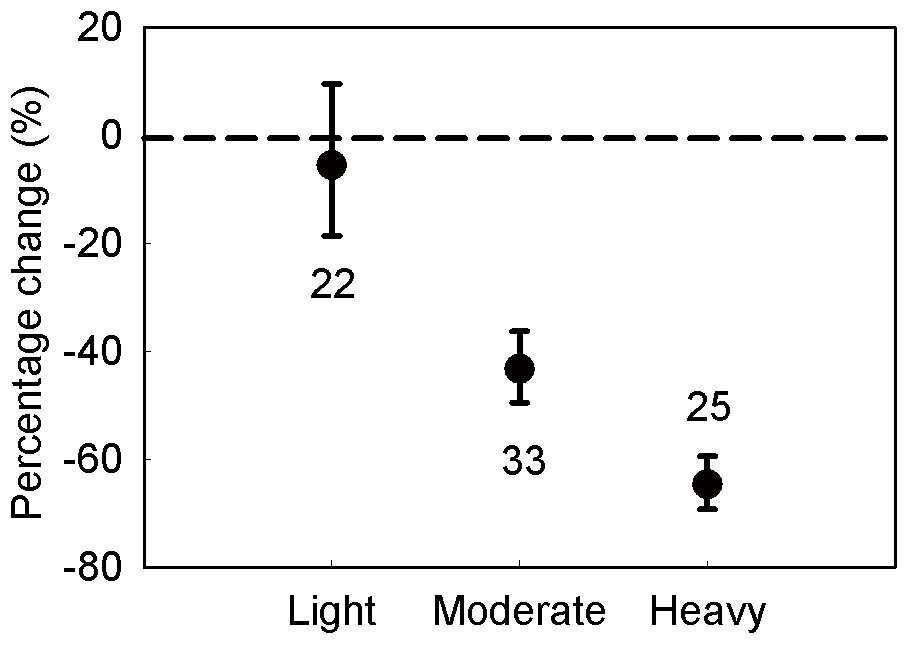
Percentage changes in aboveground biomass in response to different grazing intensities. Grazing intensity was divided into three levels: light, moderate, heavy. Values are means ± 95% CI. The number of observations used in the analysis is shown near the bar.

The bootstrap CI of percentage change in the light grazing intensity treatment overlapped zero, which meant light grazing intensity did not have a significant effects on aboveground biomass. However, aboveground biomass was significantly reduced under moderate (−43.30%) and heavy (−64.68%) grazing intensities (Figure 4).

### Response Patterns of Belowground Biomass to Grazing

#### 1. Total belowground biomass

Response patterns of belowground biomass to grazing did not show difference among grassland types (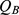 = 1.24, P = 0.74), but showed significant difference between different grazing intensities (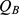 = 15.09, P<0.001).

**Figure 5:**
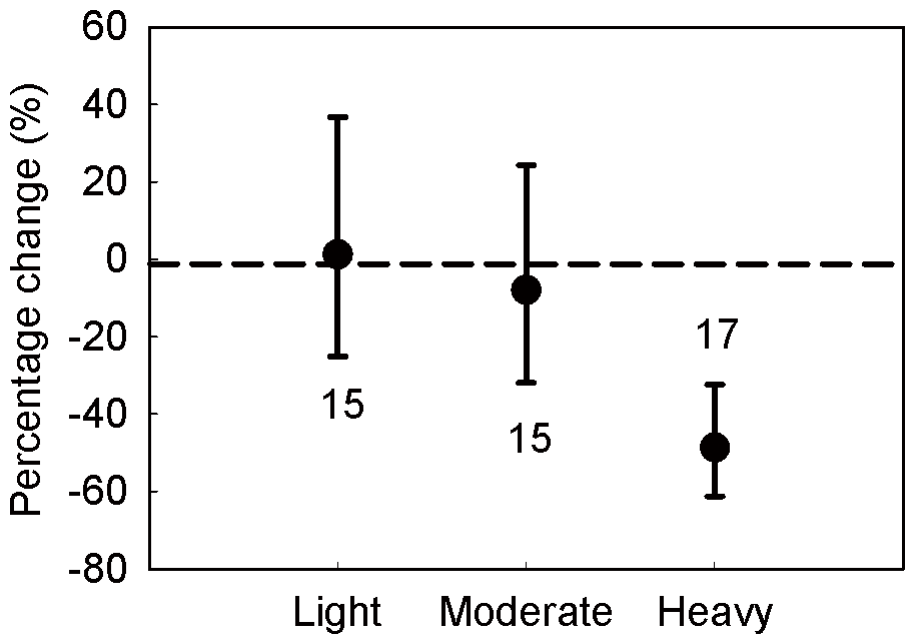
Percentage changes in belowground biomass in response to different grazing intensities. Grazing intensity was divided into three levels: light, moderate, heavy. Values are means ± 95% CI. The number of observations for each grazing intensity used in the analysis is shown near the bar.

Grazing had no significant effects at both light and moderate grazing intensities and their 95% CIs overlapped. However, belowground biomass was significantly reduced by 48.77% in heavy grazing intensity treatment (with a 95% confidence interval of −32.41%∼−61.17%; Figure 5).

#### 2. Belowground biomass of 0∼10 cm, 10∼20 cm and 20∼30 cm

**Figure 6:**
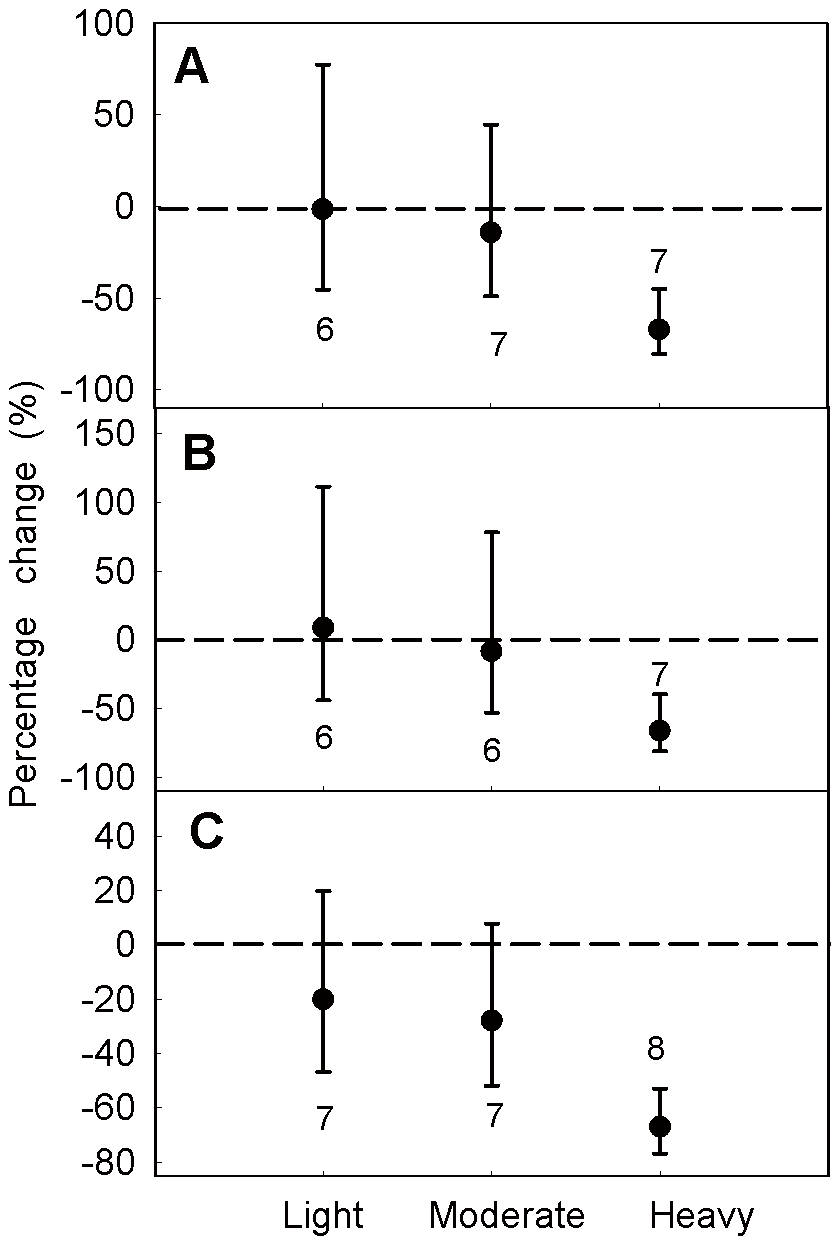
Percentage changes in belowground biomass in response to different grazing intensities. Grazing intensity was divided into three levels: light, moderate, heavy. Belowground biomass was divided into 3 layers: 0∼10 cm (A), 10∼20 cm (B), 20∼30 cm (C). Values are means ± 95% CI and number of observations used in the analysis is shown near the bar.

Light and moderate grazing intensities did not have significant effects on belowground biomass of 0∼10 cm, 10∼20 cm and 20∼30 cm, but the belowground biomass of these three layers was reduced in heavy grazing intensity treatment (Figure 6).

Response patterns of belowground biomass of 0∼10 cm, 10∼20 cm and 20∼30 cm did not show significant differences between grassland types, but showed significant differences between grazing intensities (Table 1).

**Table 1:** Between-group heterogeneity (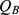) and probability (P) of grazing effects on belowground biomass of 0∼10 cm, 10∼20 cm, 20∼30 cm across different grassland types and grazing intensities.

	Categories	*Q_B_*	P
Grassland types	belowground biomass of 0∼10 cm	1.81	0.61
	belowground biomass of 10∼20 cm	6.02	0.11
	belowground biomass of 20∼30 cm	7.47	0.06
Grazing intensities	belowground biomass of 0∼10 cm	15.37	<0.01
	belowground biomass of 10∼20 cm	13.36	<0.05
	belowground biomass of 20∼30 cm	19.33	<0.001

Grassland types include typical steppe, meadow steppe, alpine steppe or desert steppe. Grazing intensity was divided into three levels: light, moderate, heavy.

### Impacts of Temperature and Precipitation on Biomass Responses to Grazing

**Figure 7:**
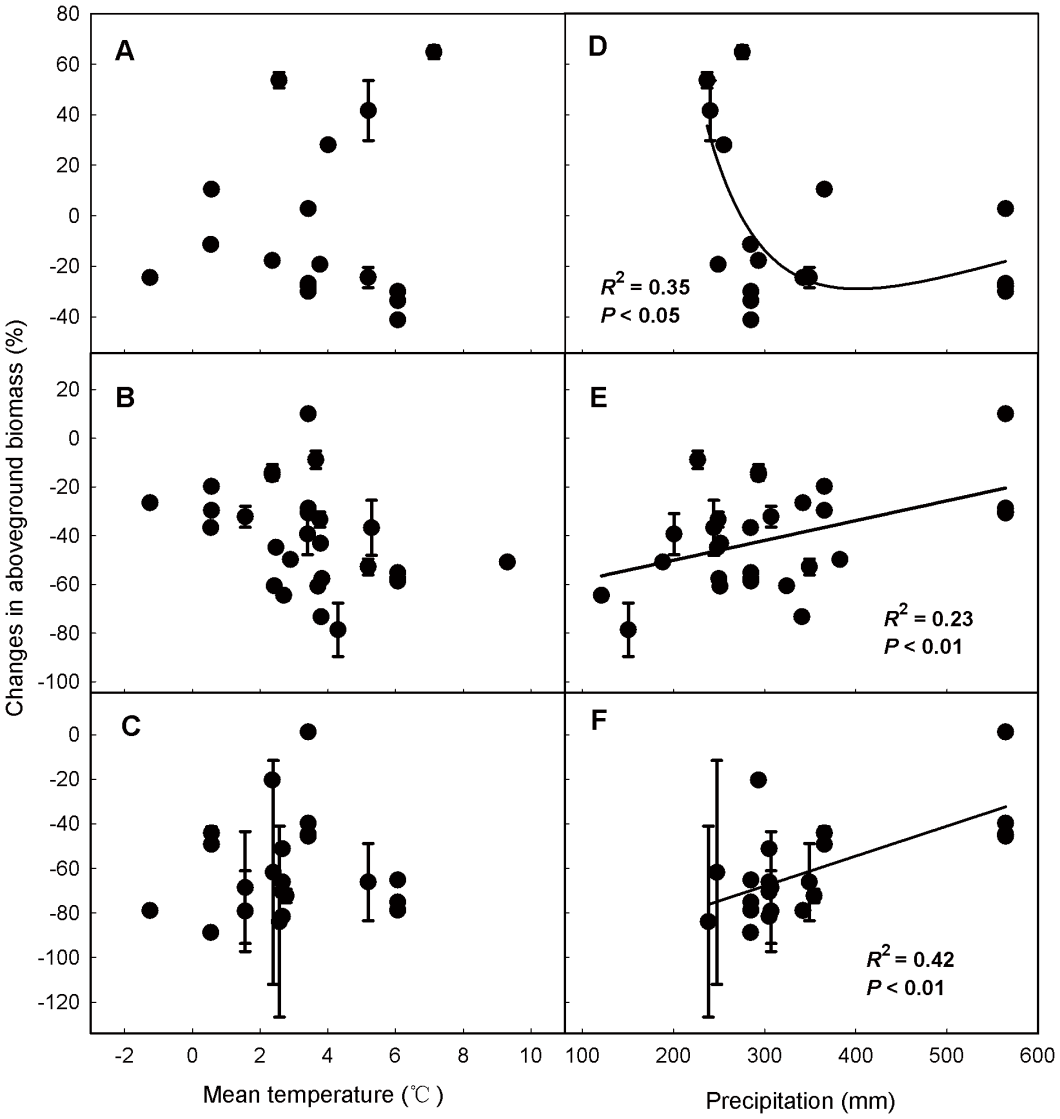
Relationships between aboveground biomass responses under different grazing intensities and mean temperature (A, B, C), precipitation (D, E, F). Grazing intensities was divided into three levels: light (A, D), moderate (B, E), heavy (C, F). Values are means ± 95% CI.

**Figure 8:**
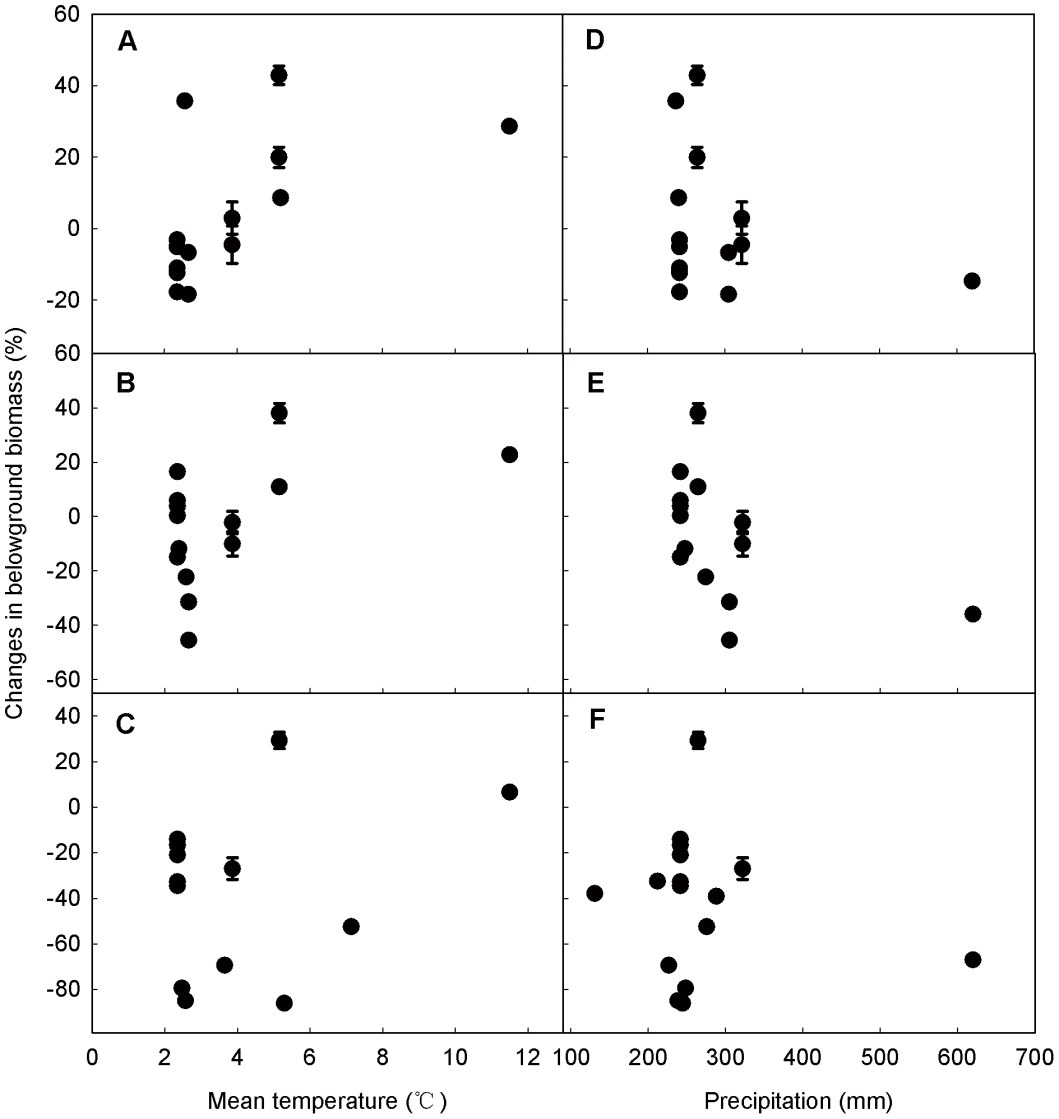
Relationships between belowground biomass responses under different grazing intensities and mean temperature (A, B, C), precipitation (D, E, F). Grazing intensities was divided into three levels: light (A, D), moderate (B, E), heavy (C, F). Values are means ± 95% CI.

Aboveground biomass responses to grazing changed with precipitation, but not with temperature (Figure 7). Response patterns of aboveground biomass to light grazing intensity showed a quadratic relationship with precipitation (*R* ^2^ = 0.35, *P*<0.05, Figure 7D), that is, with increasing precipitation, the negative effects of grazing on aboveground biomass first increased and then leveled off. In moderate and heavy grazing intensity treatment, it was a linear relationship (Moderate: *R* ^2^ = 0.23, *P*<0.01, Figure 7E; Heavy: *R* ^2^ = 0.42, *P*<0.01, Figure 7F) between aboveground biomass response patterns and precipitation of experimental year. The negative effects of grazing weakened with the increase of precipitation. However, we found no significant relationship between grazing effects on belowground biomass and either temperature or precipitation. (Figure 8).

## Discussion

Meta-analysis is a method to quantatively synthesize numerous studies to answer a certain question. The data sets used in meta-analysis are mostly collected from publications, thus publication bias is an inevitable problem we need to face and get fixed. In our analysis, 40% of whole data sets were unpublished data from transect survey. Moreover, we calculated the Failsafe number to assess the credibility of results (Nfs of AGB: 2605, BGB: 488, TB: 97, Lit: 55).

We focused on biomass rather than productivity, they are closely related in non-grazing system, but in a grazing system, it’s not the case. The measurement of productivity would be much more difficult, but productivity have great ecological means than biomass. In the future, meta-analysis on the grazing impacts on primary productivity is necessary to improve our understanding of the effects of herbivory. Here, grassland production means biomass production, and included allocation and litter.Quantitative effects of grazing on grassland production in China.

Based on 251 sets of data collected from publications and transect survey, we revealed the general response patterns of grazing effects on grassland production in China. Moreover, we considered the influence of three factors (grazing intensity, temperature, precipitation) on grazer impacts on grassland production. Milchunas & Lauenroth(1993) had quantitatively evaluated grazing effects based on data sets collected from North America, South America, Europe, Australia, Africa and Asia. Only one data set was collected from China in their study. So we took their results as global average value and compared with our results, which were average value of grazing effects in China. At the global scale, grazing may not have significant effects on biomass when took the plant as a whole, but in China, grazing has a negative effect on total biomass. The average value on aboveground biomass was higher than global average value. Belowground biomass significantly reduced, which is opposite to global average value (Table 2). This outcome indicates that grazing has higher negative effects on grassland production in China than other areas. The utilization of grasslands in China could trace back to the late Neolithic Period and utilization patterns changed with dynasty and related policy [36]. The increasing demand for natural resources and populations placed great pressures on grassland [6], 90% of grasslands in China is degraded by past overgrazing [42]. We should pay more attention to grassland degradation and desertification, seek effective management practices for sustainable use of grasslands.

**Table 2:** Differences between our research and global average value.

	Milchunas & Lauenroth(1993)	Our study
Spatial scale	Global*	China
Total biomass	No significant effect	−58.34%
Aboveground biomass	−23%	−42.77%
Belowground biomass	20%	−23.13%

* The study was based on data collected from North America, South America, Europe, Australia, Africa and Asia, but only one record was from China.

Carbon allocation can affect plant growth, community structure and function, carbon and nitrogen cycling of ecosystems [43], [44]. In our analysis the root/shoot ratio increased under grazing condition [45], [46]. Litter decomposition has great effects on the carbon cycle and nutrient turnover in terrestrial ecosystems, and this process significantly influences the development of soil, and the availability of nitrogen, phosphorus for plants and microbes. The amount of litter fall and its decomposition rate will affect ecosystem matter cycling and grassland recruitment [18], [47], [48]. Litter was reduced by grazing in our analysis.

Grazing can change the rate and status of nutrition returned to soil via feces and urine [18], [35], which may cause a fertilization effect on surface soil and thus more belowground biomass will allocate to surface soil. However, the result of paired t-test of grazing site and non-grazing site did not support this hypothesis. Whether grazing can promote root growth in surface soil depends on the original nutritional conditions of soil, grazing intensity and grazing duration.

### The Impacts of Environmental Factors on Response Patterns of Grasslands to Grazing

Plant growth is affected by heat and moisture conditions, soil fertility and disturbance such as fire and grazing [49]; plants may response differently in different heat and moisture conditions [50]. Response patterns of aboveground biomass to grazing showed significant relationships with precipitation but not with temperature. Because most of grasslands in China are distributed in semi-arid or arid areas, where water is a key limiting factor for plant growth. When lacking water, plant will be more vulnerable to grazing disturbance. In humid areas, water is not a limiting resource to plant growth, it will be more adaptable to grazing disturbance. Thus the fluctuation of precipitation in future climate change will have consequent influences on grassland ecosystem response to grazing [50], [51]. Moreover, flexible rangeland management tactics are necessary to take into consideration for balancing the demand of grassland utilization and conservation. In dry year, reducing the stocking rates is necessary for grass growth and recovery, and properly increasing the stocking rates to output more livestock products for our life in wet year.

If two grassland ecosystems have the same community biomass, it does not mean they have equal stocking capacity. Species composition may changed by grazing, especially the forage with good palatability and rich nutrition [52], [53]. We were unable to evaluate grazing impacts on good quality forage production, because they were not available in most of studies we collected. More efforts should be putted on studies conducted with the aim of revealing grazing impacts on forage production, which have great importance in sustainable use of rangeland.

Moreover, responses of grasslands to grazing are not only affected by heat and moisture conditions, but also by soil moisture and nutrient conditions [17], [54], [55], [56]. Quantitative assessments of grazing effects on soil properties, especially soil moisture, soil carbon and nitrogen content are of great significance. McSherry & Ritchie (2013) had quantitatively evaluated effects of grazing on grassland soil carbon at a global scale using meta-analysis [27], providing a detailed understanding of grazer effects on soil organic carbon, they also suggest grazers in different regions might be managed differently to help mitigate greenhouse gas emissions. Moreover, grazing could not only affect soil carbon but also affect soil water content and soil nitrogen content, which are important indicators for moisture and nutrients conditions in soil, and will influence plant growth and its responses to grazing. Thus efforts ought to be made to reveal response patterns of soil moisture and soil nitrogen to grazing and to gain a deeper insight into the role of grazing in grassland ecosystems and feedbacks. This will provide a theoretical basis for sustainable management of grassland and assessment of carbon budget.

## Supporting Information

Table S1 **The data used in the meta-analysis.** AGB: aboveground biomass; BGB: belowground biomass; BGB_U: belowground biomass of 0∼10 cm; BGB_M: belowground biomass of 10∼20 cm; BGB_L: belowground biomass of 20∼30 cm; Lit: litter; Xc: mean of the non-grazing treatment; Xe: mean of the grazing treatment; SDc: standard deviation of non-grazing treatment; SDe: standard deviation of grazing treatment; Nc: sample size of the non-grazing treatment; Ne: sample size of the grazing treatment; Type: grassland type (T: typical grassland; D: desert grassland; M: meadow grassland; A: alpine steppe); Intensity: grazing intensity (L: light; M: moderate; H: high); LAT: latitude; LONG: longitude; ALT: altitude; NA: not available.
(PDF)

Checklist S1 **PRISMA Checklist.**
(DOC)
