# Supplementary material for: Effects of Different Grazing Intensities on Grassland Production in China: A Meta-Analysis
Source: PLoS One. 2013 Dec 6;8(12):e81466. doi: 10.1371/journal.pone.0081466 (PMC3855687; doi:10.1371/journal.pone.0081466)
Supplement: Table S1 — The data used in the meta-analysis. AGB: aboveground biomass; BGB: belowground biomass; BGB_U: belowground biomass of 0∼10 cm; BGB_M: belowground biomass of 10∼20 cm; BGB_L: belowground biomass of 20∼30 cm; Lit: litter; Xc: mean of the non-grazing treatment; Xe: mean of the grazing treatment; SDc: standard deviation of non-grazing treatment; SDe: standard deviation of grazing treatment; Nc: sample size of the non-grazing treatment; Ne: sample size of the grazing treatment; Type: grassland type (T: typical grassland; D: desert grassland; M: meadow grassland; A: alpine steppe); Intensity: grazing intensity (L: light; M: moderate; H: high); LAT: latitude; LONG: longitude; ALT: altitude; NA: not available. (PDF) [file pone.0081466.s001.pdf]

Table S1 The data used in the meta-analysis

Variable	Xc	SDc	Nc	Xe	SDe	Ne	Type	Intensity	LAT	LONG	ALT	Unit
BGB_M	4.865	0.2022375	3	5.1516667	1.078162	3	T	L	43.91	116.52	1201	g/m2
BGB_L	2.8316667	0.305505	3	3.275	1.1930214	3	T	L	43.91	116.52	1201	g/m2
BGB_M	4.725	0.4821825	3	2.305	0.2457641	3	T	M	43.91	116.52	1201	g/m2
BGB_L	2.9716667	0.5819221	3	2.8683333	0.8879377	3	T	L	43.91	116.52	1201	g/m2
BGB	19.968333	0.6527123	3	18.628333	3.530118	3	T	L	43.91	116.52	1201	g/m2
BGB_L	7.5933333	0.8105142	3	4.7733333	0.5030242	3	T	M	43.91	116.52	1201	g/m2
BGB_L	3.8016667	0.9904208	3	1.545	0.2553429	3	T	M	43.91	116.52	1201	g/m2
BGB_U	9.735	1.0219589	3	6.1016667	0.41501	3	T	M	43.91	116.52	1201	g/m2
BGB_M	13.496667	1.0678171	3	9.3633333	0.6408068	3	T	M	43.91	116.52	1201	g/m2
BGB_U	12.131667	1.0950951	3	10.608333	1.7390323	3	T	L	43.91	116.52	1201	g/m2
BGB_M	4.1383333	1.1856363	3	3.9983333	0.8303212	3	T	L	43.91	116.52	1201	g/m2
BGB_U	12.405	1.353477	3	8.5416667	0.6860272	3	T	L	43.91	116.52	1201	g/m2
BGB_L	5.2316667	1.5694054	3	2.7583333	0.6066575	3	T	M	43.91	116.52	1201	g/m2
BGB_U	12.875	1.6336462	3	9.1383333	1.947109	3	T	M	43.91	116.52	1201	g/m2
BGB_M	5.8783333	1.6805455	3	4.5483333	1.4333643	3	T	M	43.91	116.52	1201	g/m2
BGB	18.261667	1.6935269	3	9.9516667	0.4212284	3	T	M	43.91	116.52	1201	g/m2
AGB	19.254667	1.6939709	3	29.586333	8.431428	3	D	L	44.11	116.32	1105	g/m2
AGB	45.946667	1.768109	3	13.536	0.5387133	3	T	H	43.91	116.52	1201	g/m2
BGB	37.156667	2.0934262	3	28.903333	0.7086842	3	T	M	43.91	116.52	1201	g/m2
BGB	11.455	2.11	3	16.37	3.365	3	D	L	41.62	110.97	1375	g/m2
BGB	11.455	2.11	3	13.74	3.08	3	D	L	41.62	110.97	1375	g/m2
BGB	11.455	2.11	3	15.82	4.235	3	D	M	41.62	110.97	1375	g/m2
BGB	11.455	2.11	3	12.71	1.645	3	D	M	41.62	110.97	1375	g/m2
BGB	11.455	2.11	3	14.81	3.92	3	D	H	41.62	110.97	1375	g/m2
BGB_U	16.066667	2.3963375	3	14.766667	0.6892266	3	T	M	43.91	116.52	1201	g/m2
BGB	19.375	2.6808394	3	15.815	1.215072	3	T	L	43.91	116.52	1201	g/m2
AGB	172.01067	2.8112235	3	58.312	1.0479943	3	T	H	43.91	116.52	1201	g/m2
Lit	90.7925	2.8857028	3	20.211	3.320014	3	NA	NA	43.53	116.55	1184	g/m2
Lit	69.733333	3.3623999	3	62.413333	0.5100327	3	NA	NA	43.91	116.52	1201	g/m2
BGB	23.985	3.4480864	3	16.445	2.9923402	3	T	M	43.91	116.52	1201	g/m2
Lit	6.772	4.332	3	36.931	4.6444971	3	NA	NA	43.73	118.95	678	g/m2
AGB	102.18667	4.3418583	3	49.872	2.4889829	3	T	H	43.91	116.52	1201	g/m2
BGB	14.88	5.125	3	14.21	2.635	3	D	L	41.62	110.97	1375	g/m2
BGB	14.88	5.125	3	15.32	1.76	3	D	L	41.62	110.97	1375	g/m2
BGB	14.88	5.125	3	13.385	1.54	3	D	M	41.62	110.97	1375	g/m2
BGB	14.88	5.125	3	14.56	0.49	3	D	M	41.62	110.97	1375	g/m2
BGB	14.88	5.125	3	10.87	1.575	3	D	H	41.62	110.97	1375	g/m2
Lit	48.843	5.359375	3	14.518	4.3538122	3	NA	NA	42.93	120.7	359	g/m2
AGB	21.641667	6.1700115	3	19.732333	3.1529923	3	D	M	44.08	113.57	972	g/m2
AGB	31.213333	6.2820549	3	51.431	9.6580157	3	D	L	42.93	120.7	359	g/m2
AGB	87.69	6.74	3	51.48	3.51	3	D	L	41.78	111.88	1450	g/m2

AGB	87.69	6.74	3	37.28	2.8	3	D	M	41.78	111.88	1450	g/m2
AGB	87.69	6.74	3	21.8	3.27	3	D	H	41.78	111.88	1450	g/m2
AGB	34.592667	7.9237138	3	19.085667	1.2266036	3	T	M	43.55	116.67	1254	g/m2
AGB	112.49067	8.8717674	3	33.253333	1.1904963	3	T	H	43.91	116.52	1201	g/m2
AGB	111.99	10.23	3	78.43	12.87	3	D	L	41.78	111.88	1450	g/m2
AGB	111.99	10.23	3	46.28	5.9	3	D	M	41.78	111.88	1450	g/m2
AGB	111.99	10.23	3	23.78	4.67	3	D	H	41.78	111.88	1450	g/m2
AGB	156.05067	10.432504	3	28.778667	1.2755929	3	T	H	43.91	116.52	1201	g/m2
Lit	20.944333	11.464	3	21.947667	20.339581	3	NA	NA	43.58	118.73	687	g/m2
AGB	53.080333	12.07163	3	33.574	17.442761	3	M	M	43.58	118.73	687	g/m2
AGB	29.465333	14.615272	3	41.730333	12.547435	3	M	L	43.73	118.95	678	g/m2
AGB	125.19	15.26	3	83.08	3.3	3	D	L	41.78	111.88	1450	g/m2
AGB	125.19	15.26	3	56.12	6.21	3	D	M	41.78	111.88	1450	g/m2
AGB	125.19	15.26	3	43.59	2.86	3	D	H	41.78	111.88	1450	g/m2
Lit	25.512	15.512314	3	3.922	0.3959798	3	NA	NA	44.08	113.57	972	g/m2
Lit	170.95633	17.662294	3	24.611	14.796698	3	NA	NA	43.55	116.67	1254	g/m2
AGB	429	20.78461	3	285	8.660254	3	A	L	33.98	102	3500	g/m2
AGB	429	20.78461	3	237	10.392305	3	A	M	33.98	102	3500	g/m2
AGB	429	20.78461	3	127	3.4641016	3	A	H	33.98	102	3500	g/m2
AGB	207.373	25.940925	3	156.682	49.886527	3	D	L	41.77	111.88	1450	g/m2
AGB	207.373	25.940925	3	97.9263	27.935728	3	D	M	41.77	111.88	1450	g/m2
AGB	207.373	25.940925	3	70.2765	47.890858	3	D	H	41.77	111.88	1450	g/m2
Lit	36.922	26.935035	3	47.648667	16.816499	3	NA	NA	44.11	116.32	1105	g/m2
Lit	41.787	27.202954	3	20.092	4.5679098	3	NA	NA	43.91	116.25	1179	g/m2
AGB	61.213333	33.858033	3	9.8686667	8.6262178	3	D	H	43.91	116.25	1179	g/m2
BGB	1905.8	38.971143	3	1625.5	36.546272	3	A	L	33.98	102	3500	g/m2
BGB	1905.8	38.971143	3	1221.7	38.971143	3	A	M	33.98	102	3500	g/m2
BGB	1905.8	38.971143	3	629.5	23.729096	3	A	H	33.98	102	3500	g/m2
AGB	54.681333	59.070195	3	20.895333	4.840928	3	D	H	43.53	116.55	1184	g/m2
BGB	2465	240.75506	3	1666	157.61662	3	T	H	43.78	116.92	1250	g/m2
BGB	2390	282.32428	3	1487	81.406388	3	T	H	43.78	116.92	1250	g/m2
BGB	2677	318.69735	3	1632	187.06149	3	T	H	43.78	116.92	1250	g/m2
BGB_L	23.18	0.18	5	20.12	0.58	5	M	L	43.73	118.95	678	g/m2
BGB_L	9.84	0.29	5	3.42	0.24	5	D	H	44.08	113.57	972	g/m2
BGB_M	47.88	0.38	5	32.78	0.91	5	M	L	43.73	118.95	678	g/m2
BGB_L	56	0.52	5	8.3	0.79	5	D	H	43.92	116.25	1179	g/m2
BGB_L	51.17	0.75	5	6.4	0.54	5	M	H	43.58	118.73	687	g/m2
BGB_M	18.36	0.88	5	7.32	0.54	5	D	H	44.08	113.57	972	g/m2
BGB_U	61.36	0.94	5	29.64	1.78	5	D	H	42.93	120.7	359	g/m2
BGB_M	114	1.05	5	23.3	0.67	5	D	H	43.92	116.25	1179	g/m2
BGB_L	15.73	1.06	5	7.32	0.22	5	D	H	42.93	120.7	359	g/m2
BGB_M	29.51	1.07	5	13.8	0.6	5	D	H	42.93	120.7	359	g/m2
BGB_L	71.19	1.11	5	45.78	0.41	5	D	M	43.53	116.55	1184	g/m2

BGB_L	305.76	1.39	5	69.19	2.61	5	T	H	43.55	116.67	1254	g/m2
BGB_M	28.38	1.43	5	51.37	2.53	5	D	L	44.11	116.32	1105	g/m2
BGB_U	61.32	1.89	5	16.74	0.89	5	D	H	44.08	113.57	972	g/m2
BGB_M	521.36	1.91	5	180.54	5.15	5	T	H	43.55	116.67	1254	g/m2
BGB	106.6	1.94	5	50.76	2.26	5	D	H	42.93	120.7	359	g/m2
BGB_L	49.5	1.97	5	27.83	1.55	5	D	L	44.11	116.32	1105	g/m2
BGB_M	85.05	1.99	5	88.41	1.26	5	D	M	43.53	116.55	1184	g/m2
BGB_U	258	2.16	5	47.6	3.37	5	M	H	43.58	118.73	687	g/m2
BGB_U	218.12	2.75	5	179.52	6.38	5	M	L	43.73	118.95	678	g/m2
BGB	89.64	2.83	5	27.48	1.12	5	D	H	44.08	113.57	972	g/m2
BGB	288.99	2.89	5	313.94	11.01	5	M	L	43.73	118.95	678	g/m2
BGB_U	1801.24	3.84	5	291.04	4.97	5	T	H	43.55	116.67	1254	g/m2
BGB	419.68	4.01	5	58.9	3.75	5	M	H	43.58	118.73	687	g/m2
BGB_M	110.08	4.17	5	4.8	0.28	5	M	H	43.58	118.73	687	g/m2
BGB_U	258.93	4.65	5	230.16	3.44	5	D	M	43.53	116.55	1184	g/m2
BGB_U	546	5.21	5	76.5	2.71	5	D	H	43.92	116.25	1179	g/m2
BGB_U	141.68	5.79	5	218.79	18.15	5	D	L	44.11	116.32	1105	g/m2
BGB	413.28	5.83	5	364.56	2.51	5	D	M	43.53	116.55	1184	g/m2
BGB	716	6.12	5	108.1	2.52	5	D	H	43.92	116.25	1179	g/m2
BGB	2619.54	6.36	5	540.77	5.33	5	T	H	43.55	116.67	1254	g/m2
BGB	219.56	8.09	5	297.99	20.39	5	D	L	44.11	116.32	1105	g/m2
AGB	81.4	31.975772	5	55.2	70.883355	5	T	M	43.53	116.67	1250	g/m2
AGB	105	53.665631	5	21.9	16.546903	5	T	H	43.53	116.67	1250	g/m2
AGB	157.2	70.2125	5	49.4	47.4046	5	T	H	43.53	116.67	1250	g/m2
Lit	2.7483333	0.3207751	6	1.94	0.4974267	6	NA	NA	43.91	116.52	1201	g/m2
AGB	188.74	17.06	6	194.03	15.21	6	M	L	45.78	131.23	55	g/m2
AGB	188.74	17.06	6	207.62	11.59	6	M	M	45.78	131.23	55	g/m2
AGB	188.74	17.06	6	191.11	19.23	6	M	H	45.78	131.23	55	g/m2
AGB	407.35	23.21	6	285.29	14.92	6	M	L	45.78	131.23	55	g/m2
AGB	407.35	23.21	6	282.28	18.73	6	M	M	45.78	131.23	55	g/m2
AGB	407.35	23.21	6	221.75	20.1	6	M	H	45.78	131.23	55	g/m2
AGB	458.67	29.34	6	330.81	25.11	6	M	L	45.78	131.23	55	g/m2
AGB	458.67	29.34	6	319.56	14.01	6	M	M	45.78	131.23	55	g/m2
AGB	458.67	29.34	6	276.37	28.17	6	M	H	45.78	131.23	55	g/m2
AGB	378.95833	39.626512	6	104.79167	42.312188	6	T	H	43.91	116.52	1201	g/m2
AGB	357.91	77.35	6	261.78	1.32	6	M	L	45.78	131.23	55	g/m2
AGB	357.91	77.35	6	255.09	21.11	6	M	M	45.78	131.23	55	g/m2
AGB	357.91	77.35	6	198.15	18.93	6	M	H	45.78	131.23	55	g/m2
BGB_U	1989.5	228.9091	6	1500.5417	189.69287	6	T	M	43.91	116.52	1201	g/m2
AGB	164.18	5.09	7	145.48	5.19	7	M	L	49.34	120.03	1100	g/m2
AGB	164.18	5.09	7	103.89	27.6	7	M	M	49.34	120.03	1100	g/m2
AGB	164.18	5.09	7	18.4	4.74	7	M	H	49.34	120.03	1100	g/m2
AGB	381.33	16.19	7	287.97	13.03	7	M	L	49.34	120.03	1100	g/m2

AGB	381.33	16.19	7	280.33	10.65	7	M	M	49.34	120.03	1100	g/m2
AGB	381.33	16.19	7	80.3	5.9	7	M	H	49.34	120.03	1100	g/m2
AGB	132.54	39.977302	7	112.65	24.446742	7	T	M	43.53	116.67	1250	g/m2
AGB	132.54	39.977302	7	109.03	7.990169	7	T	L	43.53	116.67	1250	g/m2
AGB	132.54	39.977302	7	113.98	41.723498	7	T	M	43.53	116.67	1250	g/m2
AGB	132.54	39.977302	7	105.57	21.404128	7	T	H	43.53	116.67	1250	g/m2
AGB	115.8	12.445079	8	148.3	41.860721	8	M	L	43.45	116.78	1380	g/m2
AGB	140.5	16.404877	8	59.5	13.010765	8	T	M	43.58	116.73	1190	g/m2
AGB	178	19.516147	8	69.9	9.0509668	8	T	M	43.53	116.55	1180	g/m2
AGB	97.1	22.91026	8	78.4	21.496046	8	T	L	43.62	116.67	1200	g/m2
AGB	178.2	30.829856	8	101.3	20.364675	8	T	M	43.53	116.67	1250	g/m2
AGB	574.9	113.70277	8	382.9	172.8169	8	M	M	43.62	116.68	1150	g/m2
Lit	91.5576	6.2408	10	84.1564	6.2648	10	NA	NA	37.48	101.2	3250	g/m2
Lit	91.5576	6.2408	10	78.314	14.1304	10	NA	NA	37.48	101.2	3250	g/m2
Lit	91.5576	6.2408	10	53.6232	20.4188	10	NA	NA	37.48	101.2	3250	g/m2
Lit	91.5576	6.2408	10	36.79192	12.57128	10	NA	NA	37.48	101.2	3250	g/m2
Lit	91.5576	6.2408	10	24.67868	15.70092	10	NA	NA	37.48	101.2	3250	g/m2
AGB	272.321	43.15	10	96.73	49.106	15	T	M	43.92	116.52	1201	g/m2
AGB	400.298	47.619	10	200.893	52.063	15	T	M	43.92	116.52	1201	g/m2
AGB	194.94	47.62	10	41.67	50.59	15	T	M	43.92	116.52	1201	g/m2
AGB	367.56	47.63	10	98.21	49.106	15	T	M	43.92	116.52	1201	g/m2
AGB	90.77	50.59	10	55.06	47.62	15	T	M	43.92	116.52	1201	g/m2
AGB	408.84	105.236	10	451.688	116.228	10	A	L	37.48	101.2	3250	g/m2
AGB	408.84	105.236	10	328.052	86.388	10	A	M	37.48	101.2	3250	g/m2
AGB	408.84	105.236	10	287.6612	64.1796	10	A	M	37.48	101.2	3250	g/m2
AGB	408.84	105.236	10	228.4208	103.6532	10	A	H	37.48	101.2	3250	g/m2
AGB	408.84	105.236	10	207.6776	54.974	10	A	H	37.48	101.2	3250	g/m2
AGB	2867.94	139.26	10	1768.55	167.18	15	T	M	43.92	116.52	1201	g/m2
AGB	2586.26	148.63	10	1622.06	157.96	15	T	M	43.92	116.52	1201	g/m2
AGB	1959.36	477.6304	10	962.88	312.1801	10	A	M	35.97	101.88	3500	g/m2
BGB_L	0.21	0.02	20	0.14	0.02	20	T	L	43.92	116.52	1201	g/m2
BGB_L	0.21	0.02	20	0.17	0.03	20	T	M	43.92	116.52	1201	g/m2
BGB_L	0.21	0.02	20	0.09	0.01	20	T	H	43.92	116.52	1201	g/m2
BGB_L	0.37	0.04	20	0.23	0.02	20	T	L	43.92	116.52	1201	g/m2
BGB_L	0.37	0.04	20	0.33	0.08	20	T	M	43.92	116.52	1201	g/m2
BGB_L	0.37	0.04	20	0.15	0.01	20	T	H	43.92	116.52	1201	g/m2
BGB_M	0.61	0.04	20	0.59	0.05	20	T	L	43.92	116.52	1201	g/m2
BGB_M	0.61	0.04	20	0.72	0.1	20	T	M	43.92	116.52	1201	g/m2
BGB_M	0.61	0.04	20	0.37	0.04	20	T	H	43.92	116.52	1201	g/m2
BGB	2.87	0.14	20	2.78	0.16	20	T	L	43.92	116.52	1201	g/m2
BGB	2.87	0.14	20	2.88	0.21	20	T	M	43.92	116.52	1201	g/m2
BGB	2.87	0.14	20	2.27	0.12	20	T	H	43.92	116.52	1201	g/m2
BGB_U	2.21	0.15	20	2.02	0.2	20	T	L	43.92	116.52	1201	g/m2

BGB_U	2.21	0.15	20	2.31	0.18	20	T	M	43.92	116.52	1201	g/m2
BGB_U	2.21	0.15	20	1.62	0.12	20	T	H	43.92	116.52	1201	g/m2
BGB	3.51	0.16	20	3.33	0.22	20	T	L	43.92	116.52	1201	g/m2
BGB	3.51	0.16	20	4.09	0.26	20	T	M	43.92	116.52	1201	g/m2
BGB	3.51	0.16	20	3.02	0.19	20	T	H	43.92	116.52	1201	g/m2
BGB	3.18	0.18	20	2.83	0.21	20	T	L	43.92	116.52	1201	g/m2
BGB	3.18	0.18	20	3.37	0.3	20	T	M	43.92	116.52	1201	g/m2
BGB	3.18	0.18	20	2.14	0.13	20	T	H	43.92	116.52	1201	g/m2
BGB	4.69	0.23	20	3.86	0.2	20	T	L	43.92	116.52	1201	g/m2
BGB	4.69	0.23	20	3.99	0.19	20	T	M	43.92	116.52	1201	g/m2
BGB	4.69	0.23	20	3.91	0.22	20	T	H	43.92	116.52	1201	g/m2
BGB	3.4	0.25	20	2.98	0.3	20	T	L	43.92	116.52	1201	g/m2
BGB	3.4	0.25	20	3.53	0.37	20	T	M	43.92	116.52	1201	g/m2
BGB	3.4	0.48	20	2.23	0.47	20	T	H	43.92	116.52	1201	g/m2
AGB	204.3	56.796127	20	80.5	28.62167	20	T	M	43.53	116.67	1250	g/m2
Lit	262.4	68.870894	20	28.7	28.62167	20	NA	NA	43.53	116.67	1250	g/m2
BGB_L	34.8	11.9	54	42	10.6	54	A	H	25.08	110.43	1600	g/m2
BGB_L	34.8	11.9	54	48.8	14.5	54	A	M	25.08	110.43	1600	g/m2
BGB_L	34.8	11.9	54	36.1	11.6	54	A	L	25.08	110.43	1600	g/m2
BGB_M	69.2	20.6	54	100.3	25.8	54	A	H	25.08	110.43	1600	g/m2
BGB_M	69.2	20.6	54	122.8	19.2	54	A	M	25.08	110.43	1600	g/m2
BGB_M	69.2	20.6	54	93.3	13.2	54	A	L	25.08	110.43	1600	g/m2
BGB_U	962.8	340	54	995.7	26.1	54	A	H	25.08	110.43	1600	g/m2
BGB_U	962.8	340	54	1138.5	303	54	A	M	25.08	110.43	1600	g/m2
BGB_U	962.8	340	54	1243.3	300	54	A	L	25.08	110.43	1600	g/m2
BGB	1066.8	372.5	54	1138	62.5	54	A	H	25.08	110.43	1600	g/m2
BGB	1066.8	372.5	54	1310.1	336.7	54	A	M	25.08	110.43	1600	g/m2
BGB	1066.8	372.5	54	1372.7	324.8	54	A	L	25.08	110.43	1600	g/m2

AGB: aboveground biomass; BGB: belowground biomass; BGB_U: belowground biomass of 0~10cm; BGB_M: belowground biomass of 10~20cm; BGB_L: belowground biomass of 20~30cm; Lit: litter; Xc: mean of the non-grazing treatment; Xe: mean of the grazing treatment; SDc: standard deviation of non-grazing treatment; SDe: standard deviation of grazing treatment; Nc: sample size of the non-grazing treatment; Ne: sample size of the grazing treatment; Type: grassland type (T: typical grassland; D: desert grassland; M: meadow grassland; A: alpine steppe); Intensity: grazing intensity (L: light; M: moderate; H: high); LAT: latitude; LONG: longitude; ALT: altitude; NA: not available.
